# Oncogenic UBE3C promotes breast cancer progression by activating Wnt/β-catenin signaling

**DOI:** 10.1186/s12935-020-01733-7

**Published:** 2021-01-06

**Authors:** Chen Hang, Shanojie Zhao, Tiejun Wang, Yan Zhang

**Affiliations:** 1 Department of Oncology, Wuxi Maternal and Child Health Hospital Affiliated to Nanjing Medical University, Wuxi, 214023 China; 2 Department of Gynecology and Obstetrics, Wuxi Maternal and Child Health Hospital Affiliated to Nanjing Medical University, No. 48, Huaishu Road, Wuxi, 214023 China

**Keywords:** Breast cancer, UBE3C, β-catenin, Cancer progression

## Abstract

**Background:**

Breast cancer (BrCa) is the most common female malignancy worldwide and has the highest morbidity among all cancers in females. Unfortunately, the mechanisms of BrCa growth and metastasis, which lead to a poor prognosis in BrCa patients, have not been well characterized.

**Methods:**

Immunohistochemistry (IHC) was performed on a BrCa tissue microarray (TMA) containing 80 samples to evaluate ubiquitin protein ligase E3C (UBE3C) expression. In addition, a series of cellular experiments were conducted to reveal the role of UBE3C in BrCa.

**Results:**

In this research, we identified UBE3C as an oncogenic factor in BrCa growth and metastasis for the first time. UBE3C expression was upregulated in BrCa tissues compared with adjacent breast tissues. BrCa patients with high nuclear UBE3C expression in tumors showed remarkably worse overall survival (OS) than those with low nuclear expression. Knockdown of UBE3C expression in MCF-7 and MDA-MB-453 BrCa cells inhibited cell proliferation, migration and invasion in vitro, while overexpression of UBE3C in these cells exerted the opposite effects. Moreover, UBE3C promoted β-catenin nuclear accumulation, leading to the activation of the Wnt/β-catenin signaling pathway in BrCa cells.

**Conclusion:**

Collectively, these results imply that UBE3C plays crucial roles in BrCa development and progression and that UBE3C may be a novel target for the prevention and treatment of BrCa.

## Background

Breast cancer (BrCa) has the highest morbidity among all cancers in females worldwide and may have caused more than 40,000 cancer-related deaths in the United States in 2019 [1]. Current therapeutic approaches for BrCa mainly focus on comprehensive treatment, including surgery, chemotherapy, radiotherapy, hormone therapy, and targeted therapy [2, 3]. BrCa in situ is usually not fatal to patients; however, advanced BrCa with lymph node and/or distant metastasis tends to cause life-threatening outcomes for patients [4]. Although increasing numbers of biomarkers and potential therapeutic targets for BrCa have been preliminarily explored, there is no defined means for diagnosis and prognostic assessment of BrCa [5, 6]. Thus, further exploration of novel biomarkers and the potential mechanisms of BrCa is still needed.

Ubiquitin protein ligase E3C (UBE3C), also named HECTH2, is an important regulator of proteasome function that continuously cycles on and off proteasomes and stimulates associations by ubiquitin conjugates through cooperation with USP14 [7]. UBE3C has been defined as a significant cancer-related functional protein that promotes cancer metastasis and growth in multiple cancers, including melanoma [8], renal cell carcinoma [9], and non-small cell lung cancer [10]. In BrCa, UBE3C was identified as a novel downstream molecule of ERα, and estrogen remarkably stimulates the E3 activity of UBE3C on CCNB1, which promotes BrCa progression [11]. In addition, the UBE3C-mediated malignant phenotypes of BrCa can be suppressed by miR-30a-5p [12]. However, the role of UBE3C in BrCa and its potential mechanisms have not been well characterized.

Here, we reported that UBE3C was overexpressed in BrCa tissues and associated with advanced clinical phenotypes and a poor prognosis. In addition, inhibition of UBE3C significantly suppressed BrCa cell proliferation, migration and invasion. Significantly, UBE3C notably promoted the activation of the Wnt/β-catenin pathway by promoting β-catenin nuclear accumulation. Our research reveals mechanistic insights into the UBE3C-mediated malignant phenotypes of BrCa, which provides a potential biomarker for the diagnosis and prognostic assessment of BrCa.

## Methods

### Clinical samples

A tissue microarray (TMA) containing 80 BrCa samples and corresponding normal tissue samples (BRC1602) was obtained from Superbiotek *Inc.* (Shanghai, China). Relevant clinicopathological characteristics recorded for each case were also provided by Superbiotek *Inc.* The use of the clinical samples in this research was approved by the Medicine Ethical Committee of Wuxi Maternal and Child Health Hospital affiliated with Nanjing Medical University.

### Immunohistochemistry

Immunohistochemistry (IHC) was performed on the TMA. TMA sections were deparaffinized at 55 °C for 30 min. The sections were then washed with xylene for three 5 min. The sections were rehydrated with successive washes in 100%, 90% and 70% gradient ethanol. Hydrogen peroxidase (0.3%, ZSGB-Bio, Beijing, China) was applied for 20 min to block endogenous peroxidase activity. A primary antibody against UBE3C (1:200 dilution, Cat. ab101512, Abcam) was used to visualize UBE3C expression. Previously described quantitative evaluation criteria were used [13, 14]. Briefly, the percentage of positively stained cells was scored on a 0–4 scale: 0 (< 5%), 1 (6–25%), 2 (26–50%), 3 (51–75%) and 4 (> 75%). The staining intensity was scored on a 0–3 scale: 0 (negative), 1 (weak), 2 (moderate), and 3 (strong). The immunoreactivity score (IRS) was calculated by multiplying the percentage of positive cells by the staining intensity. Immunostained sections were scanned using Aperio Digital Pathology Slide Scanners.

### Cell culture and transfection

The MCF-10A, MCF-7, and MDA-MB-453 cell lines were obtained from KeyGEN BioTECH *Inc.* (Nanjing, China). MCF-7 and MDA-MB-453 cells were maintained in RPMI-1640 medium (KeyGEN BioTECH *Inc.*) supplemented with 10% (v/v) fetal bovine serum (FBS) at 37 °C with 5% CO_2_. MCF-10A cells were cultured in DMEM/F12 (KeyGEN BioTECH *Inc.*) supplemented with 5% (v/v) horse serum, 20 ng/mL human EGF, 10 μg/mL insulin, 0.5 μg/mL hydrocortisone, penicillin, streptomycin and 100 ng/mL cholera toxin.

For subsequent assays, MCF-7 and MDA-MB-453 cells were transfected with UBE3C-siRNAs (The sequences of siRNAs for UBE3C were shown in Table 1), siRNA#NC, a UBE3C plasmid (Cloning vector: pcDNA3.1(+)-EGFP), or a control plasmid, which were synthesized by KeyGEN BioTECH *Inc.* (Nanjing, China), using Lipofectamine 2000 Reagent (Invitrogen) according to the manufacturer’s instructions.

**Table 1:** The sequences of siRNAs for UBE3C

siRNAs	Sequences
siRNA#1	5′-UGAAGAAGCUGGACACAAATT-3’
	5′-UUUGUGUCCAGCUUCUUCATT-3’
siRNA#2	5′-GGAAGAAAGAAGAAAGAGATT-3’
	5′-UCUCUUUCUUCUUUCUUCCTT-3’
siRNA#3	5′-CCAUAGAAGUUGUAGGUCATT-3’
	5′-UGACCUACAACUUCUAUGGTT-3’
siRNA#NC	5′-UUCUCCGAACGUGUCACGUTT-3’
	5′-ACGUGACACGUUCGGAGAATT-3’

### Quantitative real‑time PCR

Total RNA was extracted from BrCa cells using TRIzol reagent (Invitrogen). The primers for UBE3C mRNA reverse transcription were synthesized by KeyGEN BioTECH *Inc.* (Nanjing, China). qRT-PCR was conducted using the One Step TB Green™ PrimeScript™ RT-PCR Kit II (SYBR Green, TaKaRa). The primers used for gene amplification were as follows: UBE3C: 5′ TGGTGGCAGACTACAGGCTGAA 3′ (forward), 5′ GAGGCTGACGACATTGGCAAGG 3′ (reverse); and GAPDH: 5′ AGATCATCAGCAATGCCTCCT 3′ (forward), 5′ TGAGTCCTTCCACGATACCAA 3′ (reverse). The 2^−ΔΔCt^ method was used for mRNA expression analysis.

### Western blot analysis

Cells were placed in 35-mm dishes (6 × 10^5^ cells/dish). Forty-eight hours after transfection, all cells were harvested with lysis buffer for total protein extraction. SDS–polyacrylamide gel electrophoresis and western blot analysis were performed according to standard protocols. For nuclear and total protein extraction, a nuclear and total protein extraction kit was used (KeyGEN BioTECH *Inc.*). Primary antibodies specific for UBE3C (1:1000 dilution, Cat. ab101512, Abcam), β-catenin (1:1000 dilution, Cat. ab22656, Abcam), Cyclin D1 (1:1000 dilution, Cat. ab40754, Abcam), and MMP9 (1:1000 dilution, Cat. ab76003, Abcam) were used. The expression levels of proteins were normalized to those of GAPDH for each sample.

### CCK-8 assay

Cell Counting Kit-8 (CCK-8) assay is a simple and effective method to check that capability of cell proliferation. Forty-eight hours after transfection, cells were digested using 0.25% trypsin for 1 min and resuspended in RPMI 1640 medium containing 10% FBS. The suspended cells were seeded in a 96-well plate with the cell density adjusted to 4 × 10^4^ cells/mL (100 μL/well) and incubated at 37 °C in a constant-temperature incubator with 5% CO_2_ for 12, 24, 48, or 78 h. To each well, 10 μL CCK-8 was added, after which the plate was placed in the incubator for 2 h. The OD value of each well was measured at 450 nm with a microplate reader. Each experiment was repeated three times.

### Wound healing assay

For wound healing analysis, BrCa cells were seeded in 96-well plates (Costar, Corning, NY) and cultured to confluence. The cell monolayers were wounded by removing the culture insert and rinsed with PBS to remove cell debris. After 6 h of migration, the cells were stained with 0.2% (v/v) crystal violet for 20 min at room temperature. Images were acquired at 0 h and 6 h after wounding using a Nikon optics microscope connected to a PowerShot G10 camera (Canon, Tokyo, Japan). The migratory area was calculated by subtracting the distance between the edges of the wound at 6 h from that at 0 h.

### Boyden chamber assay

For cell invasion assays, 5 × 10^4^ cells in serum-free medium supplemented with 5 mg/mL BSA were inoculated into the upper compartments of a modified Boyden chamber (8.0 μm, Costar, Corning, NY). The polycarbonate membranes of the Boyden chambers were coated with Matrigel (BD Biosciences, Franklin Lakes, NJ). After 6 h, the invasive cells on the lower side of the Boyden chambers were fixed and stained with 0.2% crystal violet. The stained cells were imaged, and five microscopic fields per sample were randomly selected for quantification.

### Immunofluorescence

Coverslips were immersed in cell medium to allow cells to attach and grow, and then they were washed with PBS three times for 5 min each time. Paraformaldehyde (4%) was applied to fix the cells on the coverslips for 15 min at room temperature. The coverslips were washed with PBS three times for 3 min each time. Then, the coverslips were incubated with PBS containing 0.5% Triton X-100 for 5 min. Next, the cells were blocked using 5% skim milk for 1 h, after which anti-β-catenin antibody (1:200 dilution, Cat. ab22656, Abcam) and anti-UBE3C antibody (1:100 dilution, Cat. A6442, Abclonal) were added. After incubation overnight at 4 °C, the coverslips were washed with PBS once and incubated with corresponding second antibodies (Goat anti mouse IgG-FITC, Goat anti rabbit IgG-TRITC, 1:100 dilution, KeyGEN BioTECH *Inc.*)at room temperature for 1 h. Next, the coverslips were washed with PBS and stained using DAPI. After washing with PBS, the coverslips were sealed with ProLong™ Live Antifade Reagent (P36974, Thermo Fisher, USA.). Finally, the cells were observed under a fluorescence microscope.

### Statistical analysis

All statistical analyses were performed using SPSS 26.0 software (Chicago, IL). Most of the data were analyzed by Student’s t-test or one-way ANOVA followed by Dunnett’s multiple post hoc tests. All data are presented as the means ± SDs of five independent experiments if not otherwise noted. The associations between UBE3C expression levels and clinicopathological characteristics of BrCa were evaluated using Pearson’s chi-squared test. All statistical tests were two-sided, and a P value ≤ 0.05 was considered statistically significant.

## Results

### UBE3C is overexpressed in BrCa

**Fig. 1:**
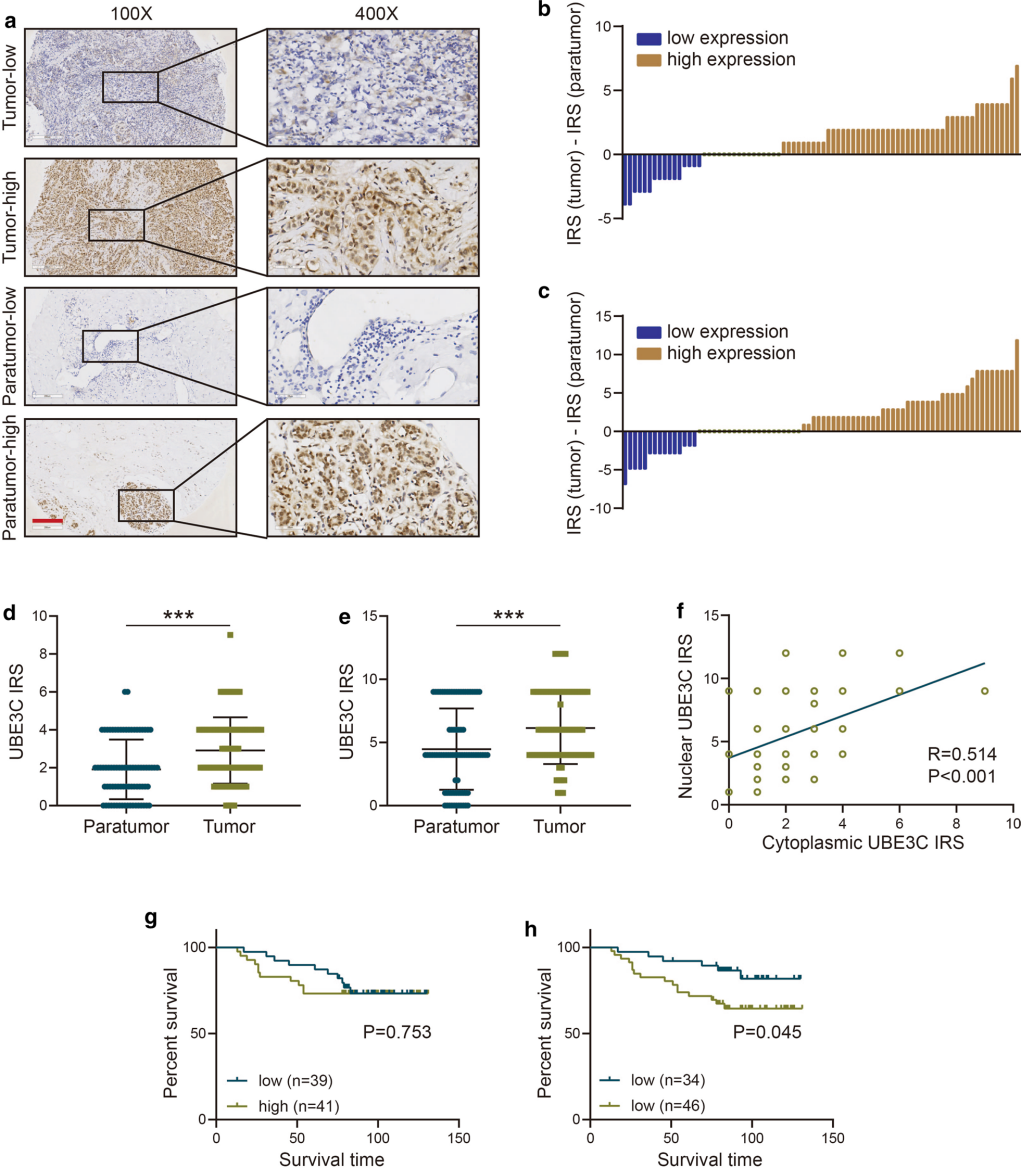
UBE3C expression in BrCa tissues. **a** Representative images showing BrCa and paired breast tissue samples stained for UBE3C. **b**, **c** The differences in cytoplasmic and nuclear UBE3C between BrCa tissues and normal tissues. **d**, **e** The relative expression levels of cytoplasmic and nuclear UBE3C in BrCa tissues compared with normal tissues. **f** Correlation between cytoplasmic and nuclear UBE3C. **g** Kaplan–Meier survival analysis of the OS of patients with BrCa stratified according to their cytoplasmic UBE3C expression level. **h** Kaplan–Meier survival analysis of the OS of patients with BrCa stratified according to their nuclear UBE3C expression level. ***P < 0.001. Bar = 200 μm

To explore the effects of UBE3C on BrCa development, the expression of UBE3C was first assessed in 80 BrCa and matched breast tissue samples. As shown in Fig. 1a, UBE3C was mainly located in the nucleus but was also expressed in the cytoplasm to some extent (Fig. 1a). We next evaluated the expression levels of UBE3C in the nucleus and cytoplasm in BrCa tissues compared with breast tissues, and the results showed that most tumor samples exhibited higher UBE3C expression in the nucleus and cytoplasm (Fig. 1b, c). In addition, compared with breast tissues, BrCa tissues showed overexpression of UBE3C in both the nucleus and cytoplasm when we used a paired Student’s t-test (Fig. 1d, e). In addition, cytoplasmic and nuclear UBE3C showed a positive correlation (Fig. 1f). Overall, similar to the expression pattern in other tumors [8–10], these results indicate that the expression of UBE3C is significantly elevated in BrCa and that upregulation of UBE3C may play an critical role in BrCa progression.

### UBE3C overexpression is associated with a poor prognosis in BrCa

We next analyzed associations between UBE3C expression and clinical parameters or prognosis in a 40-case BrCa cohort. High nuclear UBE3C expression was associated with an advanced tumor node metastasis classification (TNM) stage and a poor survival status (Additional file 1: Table S1). However, cytoplasmic UBE3C expression had no obvious associations with clinical parameters (Additional file 1: Table S1). Next, we evaluated the association between UBE3C expression and overall survival (OS) by Kaplan–Meier survival analysis with the log-rank test to assess significant differences. No significant differences were observed between the high (IRS > 2) and low (IRS ≤ 2) cytoplasmic UBE3C expression groups (Fig. 1g). However, OS was significantly shortened in the high nuclear UBE3C expression group (IRS > 4) compared to the low nuclear UBE3C expression group (IRS ≤ 4) (Fig. 1h).

### Silencing UBE3C inhibits BrCa cell proliferation, migration and invasion

**Fig. 2:**
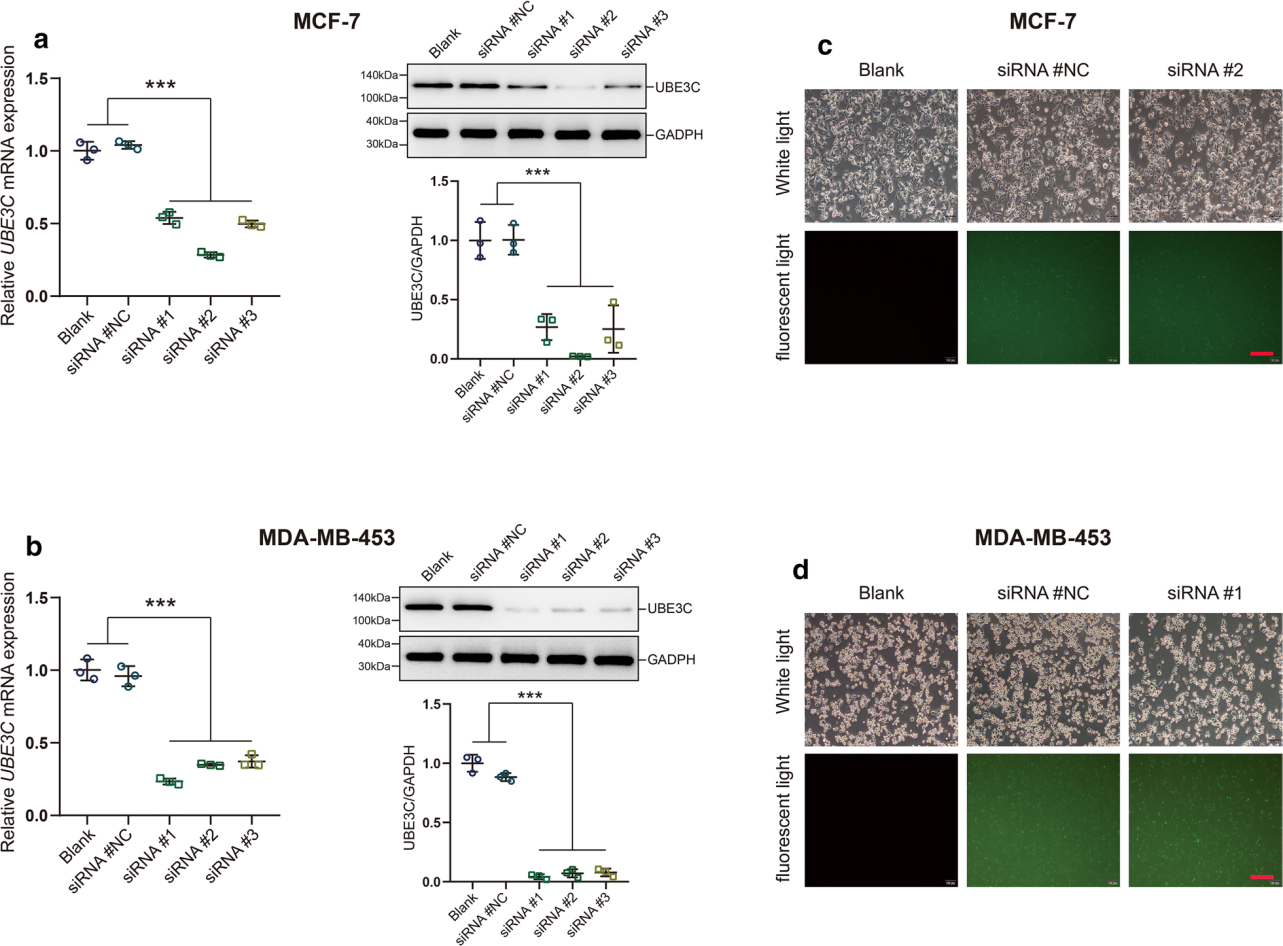
The silencing efficiency of UBE3C in BrCa cells. **a**, **b** The silencing efficiency of UBE3C in BrCa cells was confirmed by qPCR and western blotting analysis. SiRNA #2 and siRNA #1 showed highest silencing efficiency for UBE3C in MCF-7 and MDA-MB-453 cells, respectively. ***P < 0.001. **c**, **d** The silencing efficiency of UBE3C in BrCa cells and cytomorphology was determined by microscopy and immunofluorescence analysis. Green fluorescence indicated that the cells have been successfully transfected. ***P < 0.001. Bar = 200 μm

**Fig. 3:**
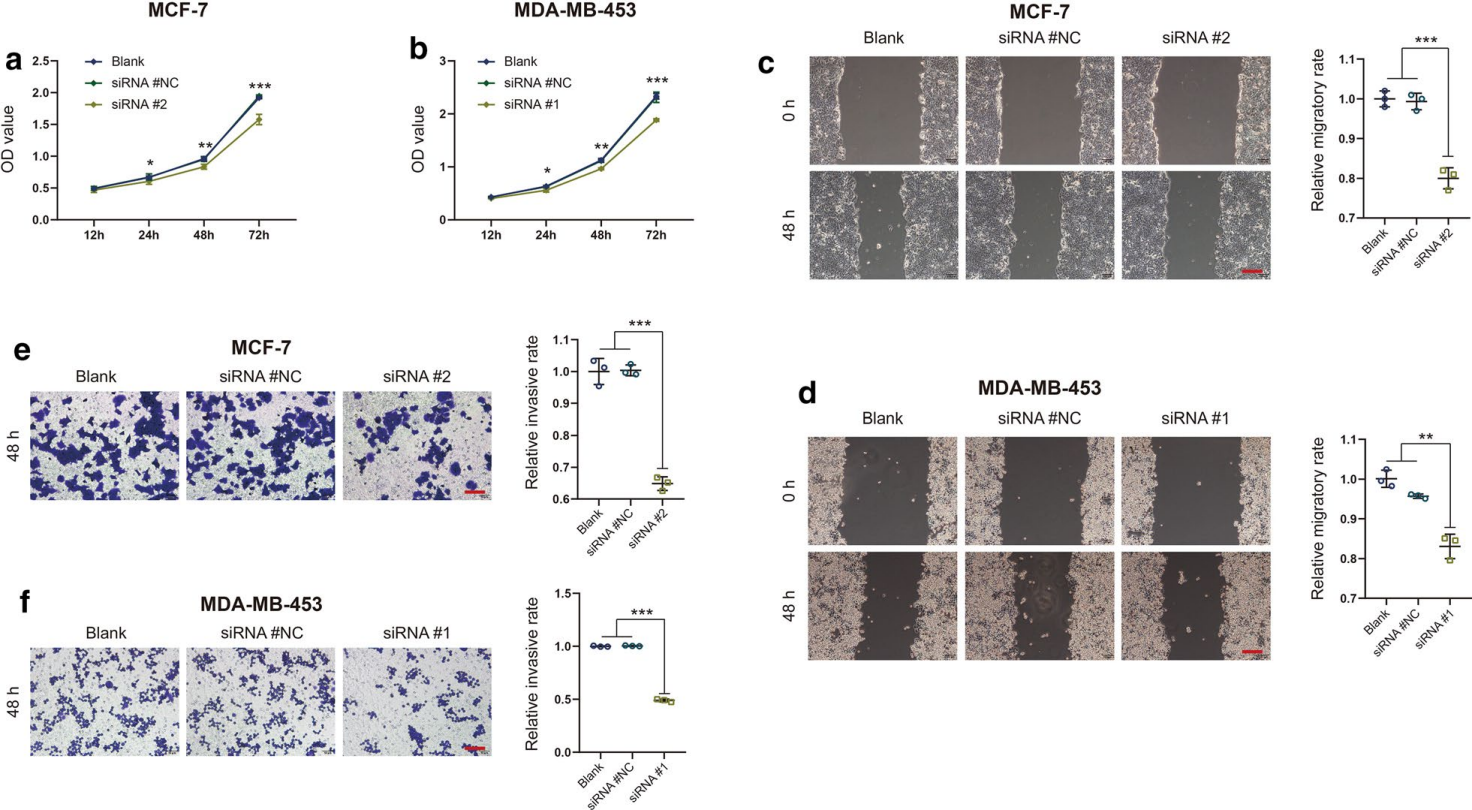
Silencing UBE3C inhibits BrCa cell proliferation, migration and invasion. **a**, **b** The proliferative capacities of control and UBE3C-silenced BrCa cells were evaluated with a CCK-8 assay. UBE3C-silenced BrCa cells showed inhibited proliferative capacities. **c**, **d** The migratory capacities of control and UBE3C-silenced BrCa cells were evaluated with a wound healing assay. UBE3C-silenced BrCa cells showed inhibited migratory capacities. **e**, **f** The invasive capacities of control and UBE3C-silenced BrCa cells were evaluated with a Boyden chamber assay. UBE3C-silenced BrCa cells showed inhibited invasive capacities. *P < 0.05, **P < 0.01, ***P < 0.001. Bar = 200 μm

Small interfering RNA (siRNA)-mediated silencing of UBE3C expression in MCF-7 and MDA-MB-453 BrCa cells was performed to assess the functional role of UBE3C in BrCa in vitro. The functional roles of UBE3C in cell proliferation, migration and invasion by BrCa cells were tested by CCK-8, wound healing and Boyden chamber assays, respectively. First, the silencing efficiency of UBE3C in BrCa cells was confirmed by qPCR, western blotting and immunofluorescence analysis (Fig. 2a–d, Additional file 2: Figure S1). Compared with corresponding control cells, UBE3C-siRNA MCF-7 and MDA-MB-453 cells showed an attenuated proliferative capacity (Fig. 3a, b). In addition, inhibition of UBE3C expression notably suppressed BrCa cell migration and invasion (Fig. 3c–f). Overall, silencing UBE3C significantly impedes BrCa progression in vitro.

### Exogenous UBE3C promotes BrCa cell proliferation, migration and invasion

**Fig. 4:**
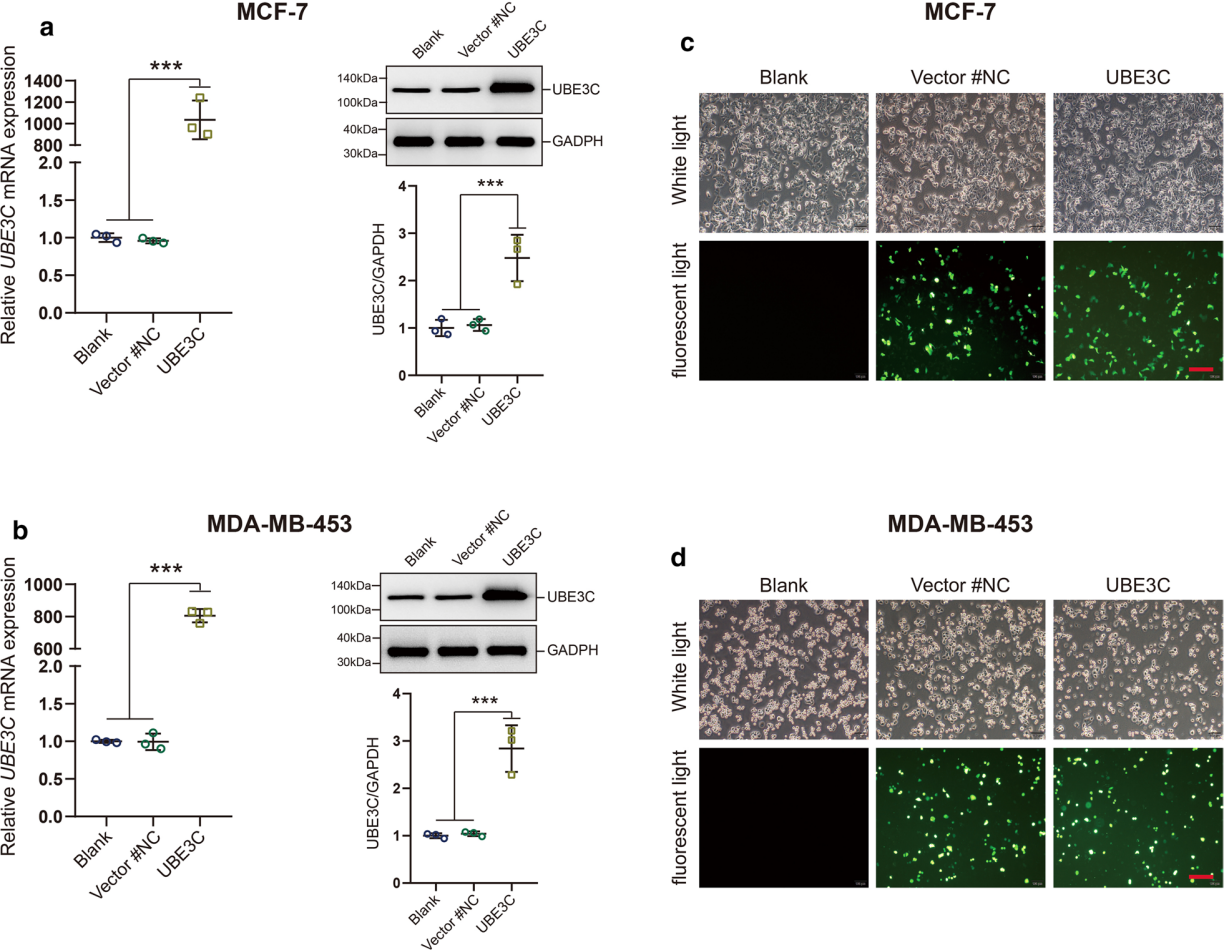
The efficiency of UBE3C overexpression in BrCa cells. **a**, **b** The efficiency of UBE3C overexpression in BrCa cells was confirmed by qPCR and western blotting analysis. UBE3C-overexpressing vector showed satisfactory transfection efficiency. **c**, **d** The efficiency of UBE3C overexpression in BrCa cells and cytomorphology was determined by microscopy and immunofluorescence analysis. Green fluorescence indicated that the cells have been successfully transfected. ***P < 0.001. Bar = 200 μm

**Fig. 5:**
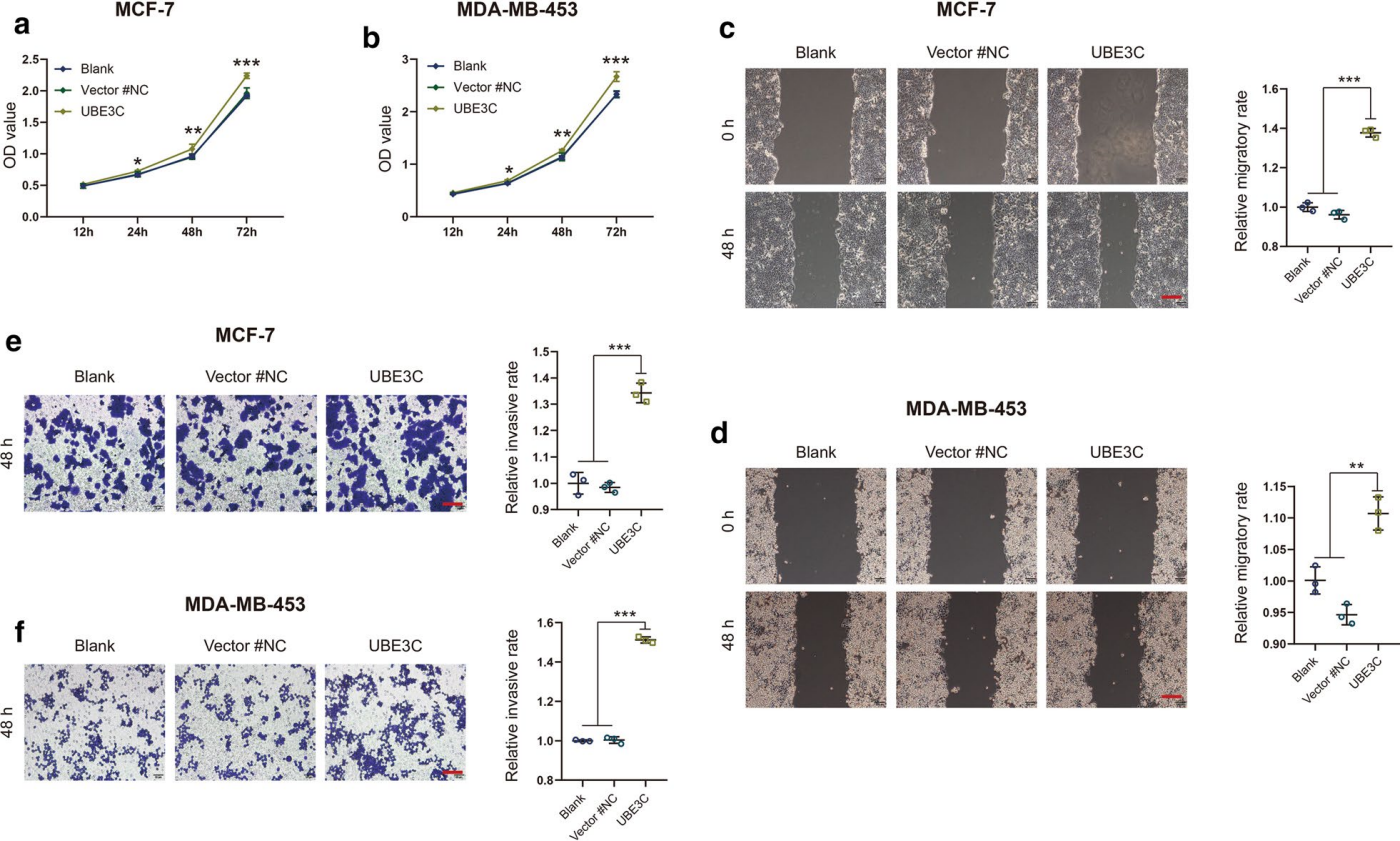
Overexpression of UBE3C promotes BrCa cell proliferation, migration and invasion. **a**, **b** The proliferative capacities of control and UBE3C-overexpressing BrCa cells were evaluated with a CCK-8 assay. UBE3C-overexpressing BrCa cells showed motivated proliferative capacities. **c**, **d** The migratory capacities of control and UBE3C-overexpressing BrCa cells were evaluated with a wound healing assay. UBE3C-overexpressing BrCa cells showed motivated migratory capacities. **e**, **f** The invasive capacities of control and UBE3C-overexpressing BrCa cells were evaluated with a Boyden chamber assay. UBE3C-overexpressing BrCa cells showed motivated invasive capacities. *P < 0.05, **P < 0.01, ***P < 0.001. Bar = 200 μm

Next, we assessed the changes in cellular behavior induced by exogenous UBE3C overexpression. The transfection efficiency of UBE3C in MCF-7 and MDA-MB-453 BrCa cells was confirmed by qPCR, western blotting and immunofluorescence analysis (Fig. 4a–d, Additional file 2: Figure S1). Compared with that of corresponding control cells, the proliferative capacity of UBE3C-overexpressing MCF-7 and MDA-MB-453 cells was significantly enhanced (Fig. 5a, b). In addition, exogenous expression of UBE3C remarkably increased BrCa cell migration and invasion (Fig. 5c–f). Overall, overexpression of UBE3C plays an oncogenic role in BrCa cells, which may be a potential target for BrCa therapy.

### UBE3C regulates the Wnt/β-catenin pathway in BrCa cells

**Fig. 6:**
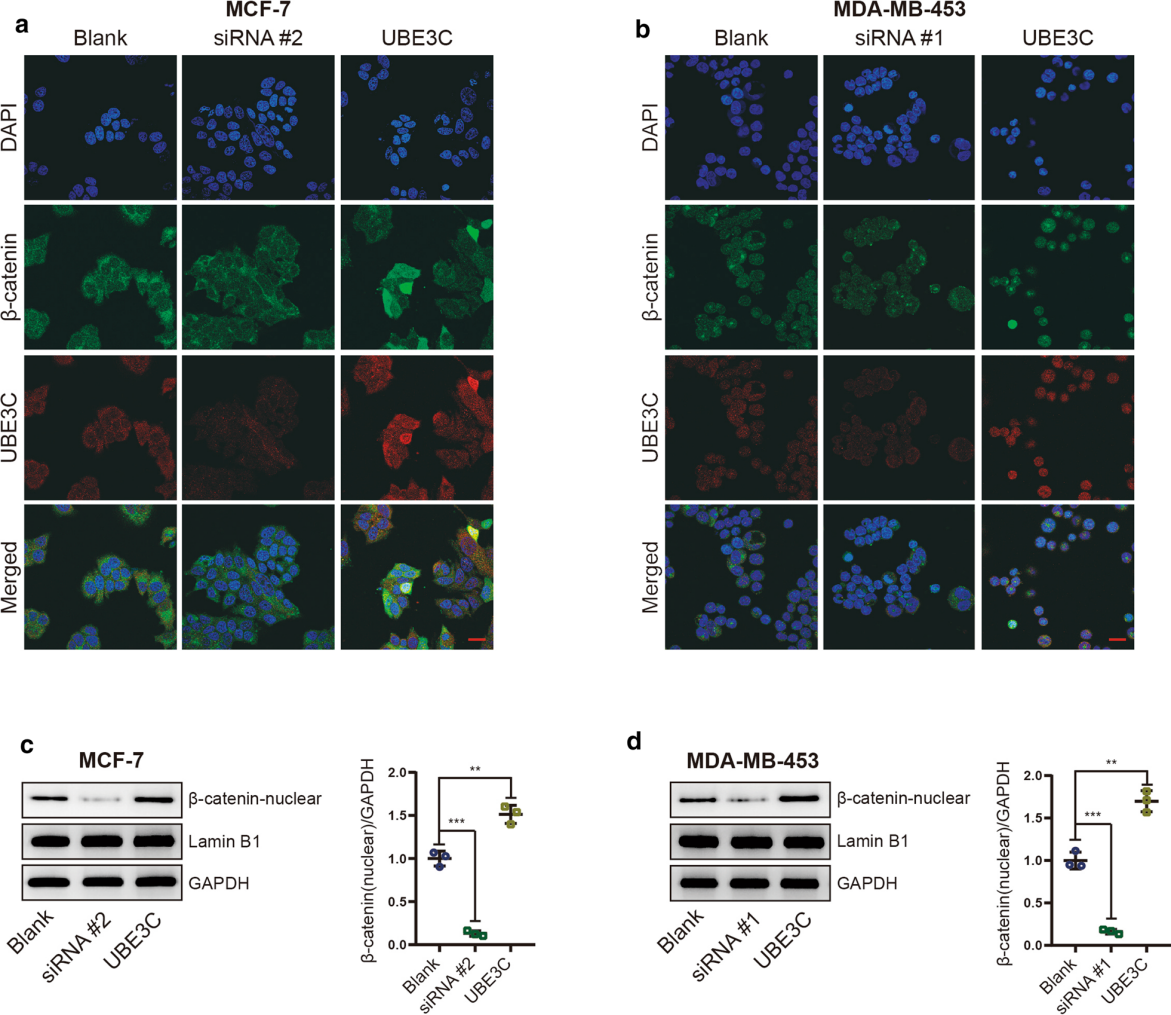
UBE3C promotes the nuclear accumulation of β-catenin. **a**, **b** Immunofluorescence staining shows that silencing or overexpressing UBE3C in BrCa cells inhibited or increased the nuclear accumulation of β-catenin, respectively. Blue: DAPI, green: β-catenin, red: UBE3C. Bar = 20 μm. **c**, **d** The expression levels of total and nuclear β-catenin in UBE3C-silenced and/or UBE3C-overexpressing BrCa cells was evaluated by western blotting. UBE3C-silenced BrCa cells showed low nuclear β-catenin level, but UBE3C-overexpressing BrCa cells were opposite. **P < 0.01, ***P < 0.001

**Fig. 7:**
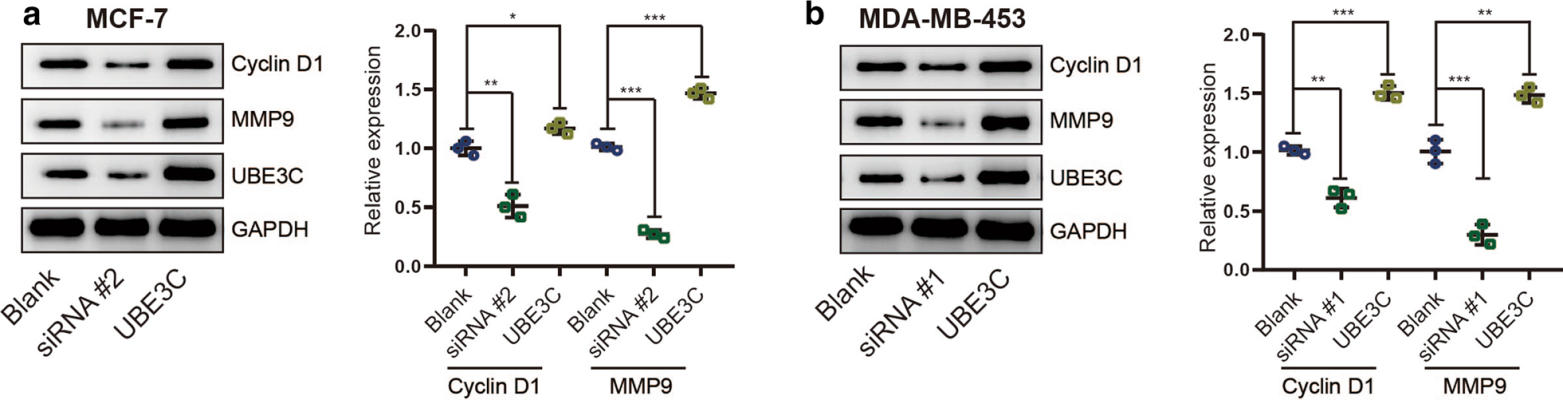
UBE3C regulates expression of Cyclin D1 and MMP9 in BrCa cell. **a**, **b** The expression levels of UBE3C, Cyclin D1 and MMP9 in UBE3C-silenced and/or UBE3C-overexpressing BrCa cells were evaluated by western blotting. UBE3C-silenced BrCa cells showed low Cyclin D1 and MMP9 levels, but UBE3C-overexpressing BrCa cells were opposite. *P < 0.05, **P < 0.01, ***P < 0.001

The canonical Wnt pathway, namely, the Wnt/β-catenin pathway, is one of the pathways most involved in the oncogenesis and progression of BrCa [15–17]. We postulated that UBE3C regulates the Wnt/β-catenin signaling pathway to mediate tumor growth and metastasis in BrCa cells. Nuclear accumulation of β-catenin is the central event in activation of the Wnt/β-catenin signaling pathway, and whether UBE3C can regulate β-catenin nuclear accumulation in BrCa cells was first investigated. Silencing UBE3C decreased the nuclear β-catenin level, while overexpressing UBE3C enhanced the nuclear β-catenin level in MCF-7 and MDA-MB-453 BrCa cells (Fig. 6a–d, Additional file 3: Figure S2). Besides, the results from immunofluorescence show that UBE3C and β-catenin is mainly located in nuclear and have positive correlation. To test whether UBE3C influences the expression levels of molecules downstream of the Wnt/β-catenin signaling pathway, we next evaluated MMP9 and Cyclin D1 expression, and the results showed that silencing UBE3C inhibited their expression and that exogenous UBE3C had the opposite effects (Fig. 7a, b, Additional file 4: Figure S3). Taken together, these data suggest that UBE3C can regulate β-catenin nuclear accumulation in BrCa cells, which may be the critical mechanism promoting BrCa progression.

## Discussion

Ubiquitination has been a research hotspot in recent years. Ubiquitin ligase E3 directly mediates the specific binding of ubiquitin to substrates and may be involved in the regulation of multiple signaling pathways, which is essential for critical cellular processes [18–20]. Ubiquitin ligases are mainly classified into two types: homologous to E6AP C-terminus (HECT) and really interesting new gene (RING) [21]. UBE3C is a member of the HECT family, and proteins in this family contain two main protein domains: an IQ motif and a HECT domain [21]. The IQ motif mediates substrate targeting, and the HECT domain binds to ubiquitin-conjugating enzymes (E2) and mediates ubiquitin conjugation to the target substrate [22]. Therefore, the HECT domain of UBE3C provides E3 ligase catalytic activity.

E3 ligases play critical roles in the regulation of multiple biological signals via selectively binding to their specific protein substrates [18, 23]. Dysregulation of the HECT E3 ligase contributes to various pathological disorders, including human cancers [24–26]. E6AP, the first identified member of the HECT E3 family [27], targets the tumor suppressor TP53 through ubiquitin-mediated proteolysis, which enhances the degradation of the TP53 protein and leads to the progression of various human cancers [24]. UBE3C, another member of the HECT E3 family, has been reported to play a critical role in multiple cancers [8–10]. However, there are currently no reports on the association between UBE3C and the progression of BrCa.

In this study, we found that UBE3C expression was significantly upregulated in BrCa tissues compared with adjacent breast tissues. In addition, high nuclear UBE3C expression was associated with an advanced TNM stage and a poor survival status. In addition, we confirmed that BrCa patients with high nuclear UBE3C expression in tissues showed worse OS than those with low nuclear UBE3C expression but that high cytoplasmic UBE3C expression did not produce a similar effect. Taken together, these results suggest that nuclear UBE3C protein expression may be a novel diagnostic and prognostic biomarker for BrCa.

In addition to the diagnostic and prognostic value of UBE3C, several scholars have found that targeting UBE3C is a promising therapeutic strategy to limit cancer progression [12, 28]. In this study, we confirmed that targeting UBE3C expression using siRNA significantly inhibited the proliferation, migration and invasion of BrCa MCF-7 and MDA-MB-453 cells. However, exogenous overexpression of UBE3C endowed BrCa MCF-7 and MDA-MB-453 cells with a stronger malignant potential.

The Wnt/β-catenin signaling pathway has been reported to be related to the oncogenesis and progression of multiple kinds of human cancers [15–17]. β-catenin is considered a positive regulator of this pathway that functions by promoting the transcription of target genes [29]. Some target genes, such as MMP9 and cyclin D1, are involved in oncogenesis, regulating cancer proliferation and metastasis [30, 31]. To the best of our knowledge, there is no research on the association between UBE3C and regulation of the Wnt/β-catenin pathway in BrCa. In this study, we found that silencing UBE3C decreased the nuclear β-catenin level, while overexpressing UBE3C enhanced the nuclear β-catenin level in MCF-7 and MDA-MB-453 BrCa cells. In addition, silencing UBE3C inhibited MMP9 and Cyclin D1 expression, and exogenous UBE3C had the opposite effects. Altogether, these results demonstrate that UBE3C may be associated with cell proliferation, migration and invasion in BrCa by mediating the Wnt/β-catenin signaling pathway. In gastric cancer, Zhang et al*.* demonstrated that UBE3C promotes gastric cancer progression through activating the β-catenin signaling via degradation of AXIN1 [32], which explained the potential mechanism of UBE3C regulating Wnt/β-catenin signaling pathway.

## Conclusion

Overall, the present study evaluated the possibility of using UBE3C as a potential biomarker for disease diagnosis, as well as identifying UBE3C as a promising indicator of the prognosis of patients with BrCa, for the first time. In addition, in agreement with clinical findings, in vitro cell experiments confirmed that UBE3C enhances BrCa cell proliferation, migration and invasion by mediating the Wnt/β-catenin signaling pathway. Overall, UBE3C plays critical roles in BrCa carcinogenesis and progression, and UBE3C may be a novel target for BrCa therapy.

## Supplementary Information

**Additional file 1: Table S1.** Association between UBE3C expression and patients’ clinical parameters in BrCa.

**Additional file 2: Figure S1.** The original image for western blotting in Fig. 2 and Fig. 4.

**Additional file 3: Figure S2.** The original image for western blotting in Fig. 6.

**Additional file 4: Figure S3.** The original image for western blotting in Fig. 7.

## Data Availability

All data are included in the article.

## Abbreviations

BrCa: Breast cancer
UBE3C: Ubiquitin protein ligase E3C
TMA: Tissue microarray
IHC: Immunohistochemistry
IRS: Immunoreactivity score
FBS: Fetal bovine serum
CCK-8: Cell Counting Kit-8
TNM: Tumor node metastasis classification
OS: Overall survival
siRNA: Small interfering RNA
RING: Really interesting new gene
HECT: Homologous to E6AP C-terminus
E2: Ubiquitin-conjugating enzymes
