# Supplementary material for: Oncogenic UBE3C promotes breast cancer progression by activating Wnt/β-catenin signaling
Source: Cancer Cell Int. 2021 Jan 6;21:25. doi: 10.1186/s12935-020-01733-7 (PMC7789303; doi:10.1186/s12935-020-01733-7)
Supplement: Supplementary file 1 — Additional file 1: Table S1. Association between UBE3C expression and patients’ clinical parameters in BrCa. [file 12935_2020_1733_MOESM1_ESM.docx]

**Table S1. Association between UBE3C expression and patients’ clinical parameters in BrCa.**

| Clinical parameters | | Case | Cytoplasm expression | | χ^2^ | P value | Nucleus expression | | χ^2^ | P value |
| --- | --- | --- | --- | --- | --- | --- | --- | --- | --- | --- |
|  |  |  | Low | High |  |  | Low | High |  |  |
| Age | ≤50 | 40 | 21 | 19 | 0.450 | 0.502 | 20 | 20 | 1.841 | 0.175 |
|  | >50 | 40 | 18 | 22 |  |  | 14 | 26 |  |  |
| T stgae | T1 | 36 | 18 | 18 | 0.041 | 0.840 | 17 | 19 | 0.597 | 0.440 |
|  | T2-4 | 44 | 21 | 23 |  |  | 17 | 27 |  |  |
| N stage | N0 | 47 | 21 | 26 | 0.755 | 0.385 | 21 | 26 | 0.222 | 0.638 |
|  | N1-3 | 33 | 18 | 15 |  |  | 13 | 20 |  |  |
| TNM stage | I-II | 59 | 30 | 29 | 0.396 | 0.529 | 29 | 30 | 4.070 | **0.044** |
|  | III | 21 | 9 | 12 |  |  | 5 | 16 |  |  |
| Tumor location | Right | 42 | 21 | 21 | 0.055 | 0.814 | 16 | 26 | 0.702 | 0.402 |
|  | Left | 38 | 18 | 20 |  |  | 18 | 20 |  |  |
| PR status | Negative | 42 | 20 | 22 | 0.045 | 0.832 | 18 | 24 | 0.005 | 0.946 |
|  | Positive | 38 | 19 | 19 |  |  | 16 | 22 |  |  |
| ER status | Negative | 28 | 16 | 12 | 1.214 | 0.270 | 14 | 14 | 0.992 | 0.319 |
|  | Positive | 52 | 23 | 29 |  |  | 20 | 32 |  |  |
| ERBB2 status | Negative | 38 | 17 | 21 | 0.467 | 0.495 | 16 | 22 | 0.005 | 0.946 |
|  | Positive | 42 | 22 | 20 |  |  | 18 | 24 |  |  |
| P53 status | Negative | 38 | 16 | 22 | 1.279 | 0.258 | 19 | 19 | 1.666 | 0.197 |
|  | Positive | 42 | 23 | 19 |  |  | 15 | 27 |  |  |
| Ki-67 status | Negative | 33 | 17 | 16 | 0.105 | 0.746 | 17 | 16 | 1.661 | 0.197 |
|  | Positive | 46 | 22 | 24 |  |  | 17 | 29 |  |  |
| OS status | Alive | 59 | 29 | 30 | 0.015 | 0.904 | 29 | 30 | 4.070 | **0.044** |
|  | Dead | 21 | 10 | 11 |  |  | 5 | 16 |  |  |
