# Supplementary material for: Oncogenic UBE3C promotes breast cancer progression by activating Wnt/β-catenin signaling
Source: Cancer Cell Int. 2021 Jan 6;21:25. doi: 10.1186/s12935-020-01733-7 (PMC7789303; doi:10.1186/s12935-020-01733-7)
Supplement: Supplementary file 2 — Additional file 2: Figure S1. The original image for western blotting in Fig. 2 and Fig. 4. [file 12935_2020_1733_MOESM2_ESM.docx]

Figure S1. The original image for western blotting in Figure 2 and Figure 4.


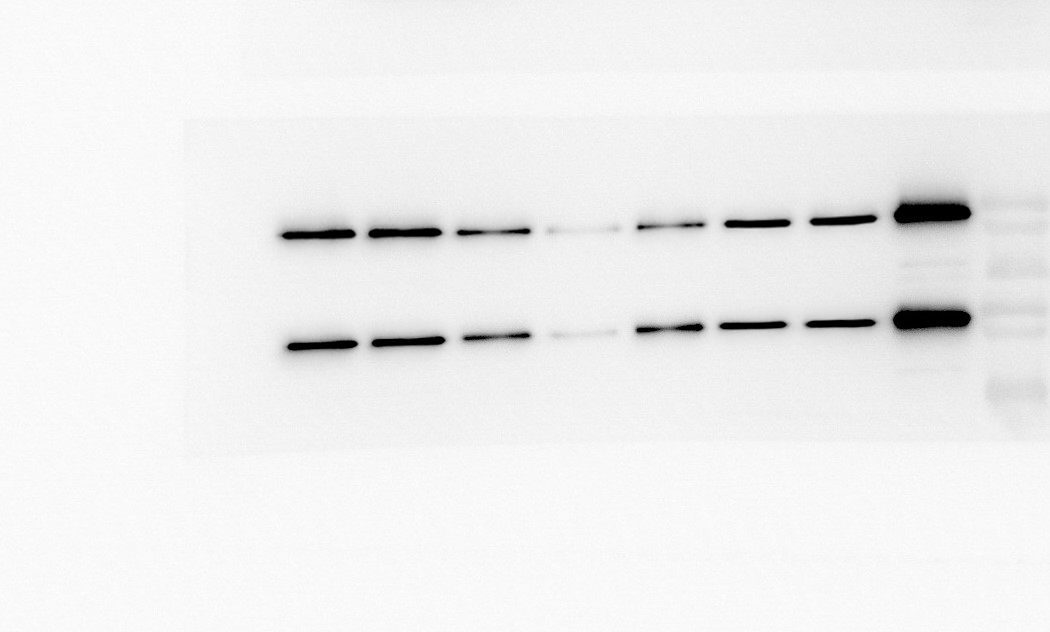


MCF-7 cell, UBE3C (Repeat 1, 2)

Blank, siRNA#NC, siRNA#1, siRNA#2, siRNA#3, Blank, Vector NC, UBE3C


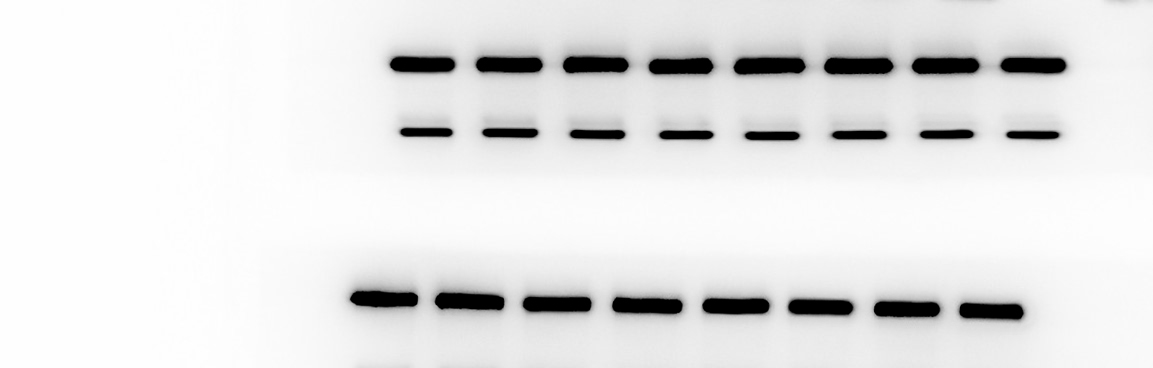


MCF-7 cell, GAPDH (Repeat 1, 2, 3)

Blank, siRNA#NC, siRNA#1, siRNA#2, siRNA#3, Blank, Vector NC, UBE3C


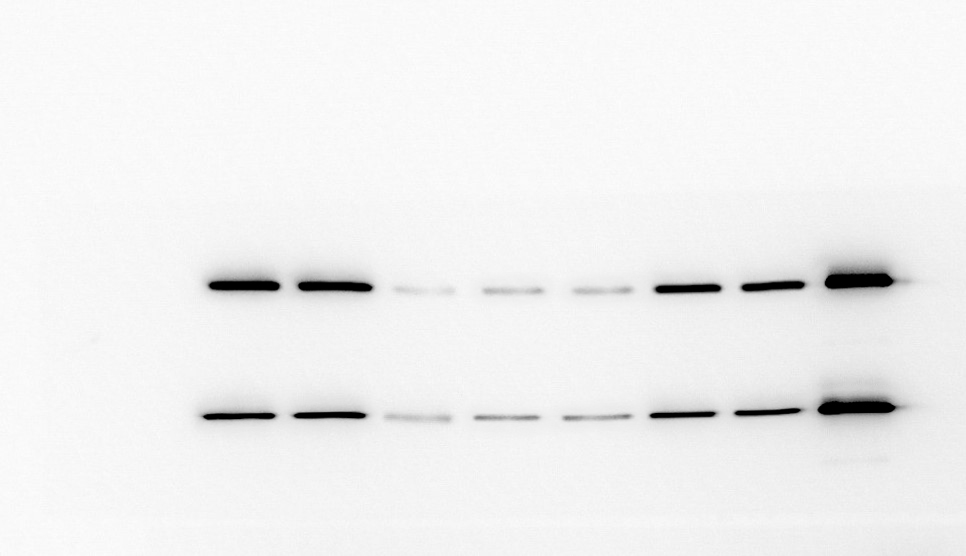


MDA-MB-453 cell, UBE3C (Repeat 1, 2)

Blank, siRNA#NC, siRNA#1, siRNA#2, siRNA#3, Blank, Vector NC, UBE3C


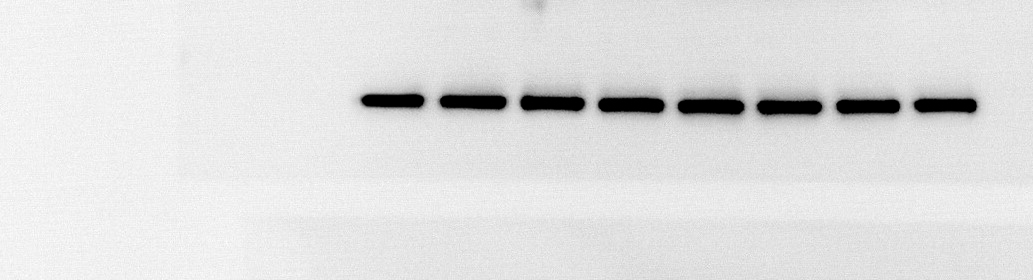


MDA-MB-453 cell, GAPDH

Blank, siRNA#NC, siRNA#1, siRNA#2, siRNA#3, Blank, Vector NC, UBE3C
