# Supplementary material for: Oncogenic UBE3C promotes breast cancer progression by activating Wnt/β-catenin signaling
Source: Cancer Cell Int. 2021 Jan 6;21:25. doi: 10.1186/s12935-020-01733-7 (PMC7789303; doi:10.1186/s12935-020-01733-7)
Supplement: Supplementary file 4 — Additional file 4: Figure S3. The original image for western blotting in Fig. 7. [file 12935_2020_1733_MOESM4_ESM.docx]

Figure S3. The original image for western blotting in Figure 7.


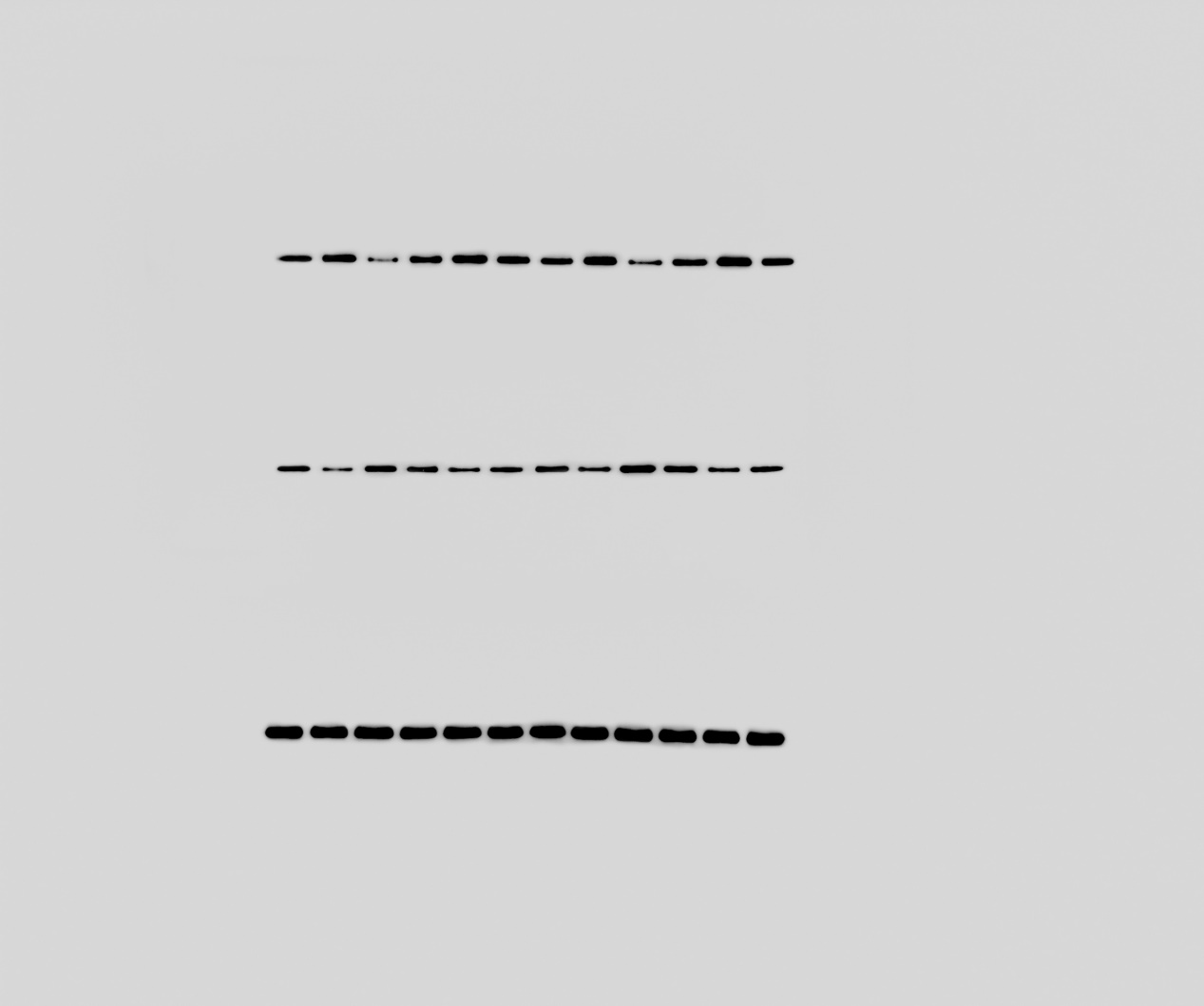


Left: MCF7 cell, right: MDA-MB-453 cell

Upper: P53# , median: CyclinD1, lower: GAPDH

MCF7 cell: Blank, siRNA-UBE3C, UBE3C, (Blank, miR-30, miR-30+UBE3C)#; MDA-MB-453 cell: Blank, siRNA-UBE3C, UBE3C, (Blank, miR-30, miR-30+UBE3C)#

# not included in this research


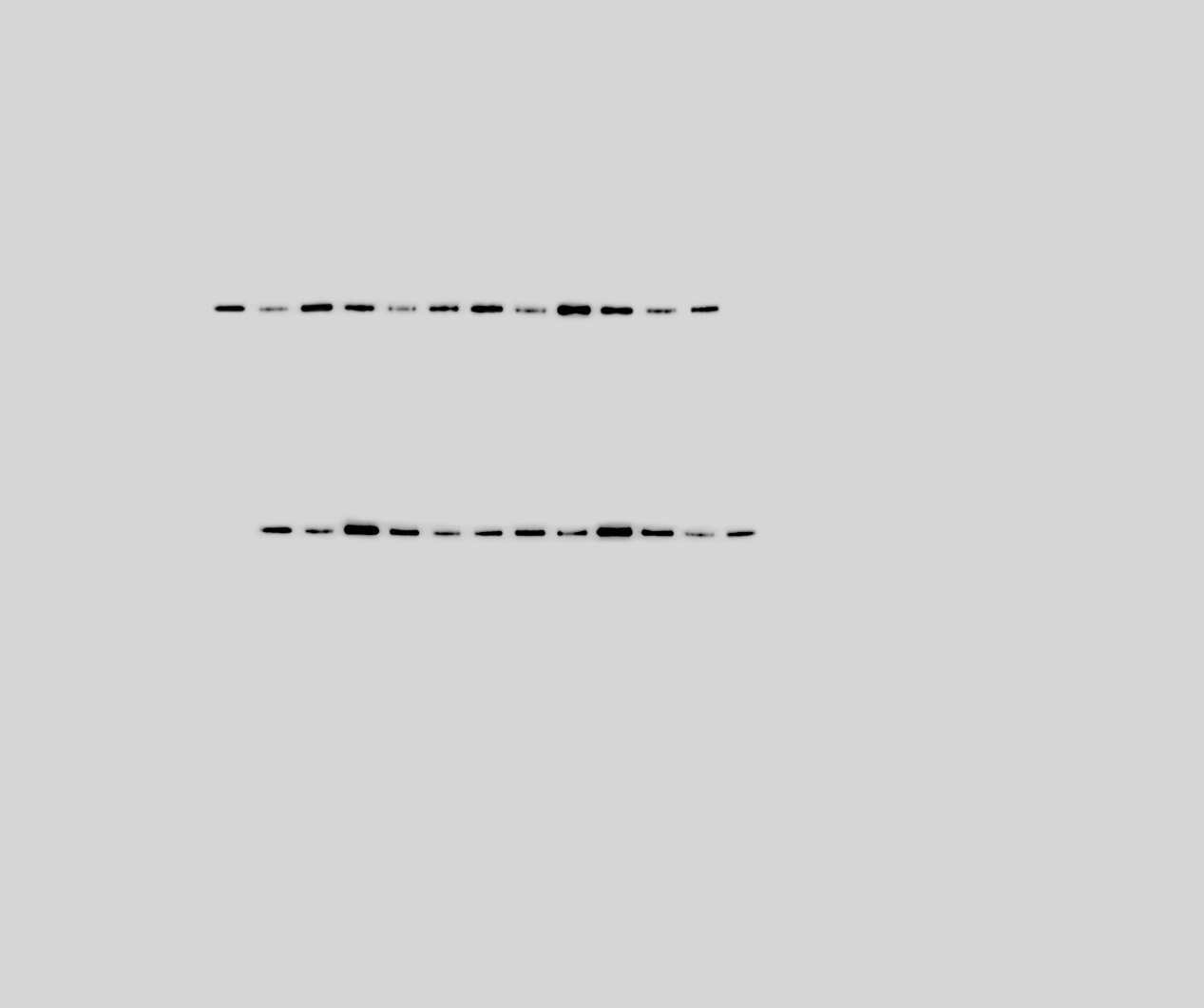


Left: MCF7 cell, right: MDA-MB-453 cell

Upper: MMP9 , lower: UBE3C

MCF7 cell: Blank, siRNA-UBE3C, UBE3C, (Blank, miR-30, miR-30+UBE3C)#; MDA-MB-453 cell: Blank, siRNA-UBE3C, UBE3C, (Blank, miR-30, miR-30+UBE3C)#

# not included in this research
